# Ethnic disparities in progression rates for sight-threatening diabetic retinopathy in diabetic eye screening: a population-based retrospective cohort study

**DOI:** 10.1136/bmjdrc-2023-003683

**Published:** 2023-11-10

**Authors:** Abraham Olvera-Barrios, Christopher G Owen, John Anderson, Alasdair N Warwick, Ryan Chambers, Louis Bolter, Yue Wu, Roshan Welikala, Jiri Fajtl, Sarah A Barman, Paolo Remagnino, Emily Y Chew, Frederick L Ferris, Aroon D Hingorani, Reecha Sofat, Aaron Y Lee, Catherine Egan, Adnan Tufail, Alicja R Rudnicka, the ARIAS Research Group, John Anderson

**Affiliations:** 1 NIHR Biomedical Research Centre, Moorfields Eye Hospital NHS Foundation Trust, London, UK; 2 Institute of Ophthalmology, University College London, London, UK; 3 Population Health Research Institute, St. George's University of London, London, UK; 4 Diabetes, Homerton Healthcare NHS Foundation Trust, London, UK; 5 Institute of Cardiovascular Science, University College London, London, UK; 6 Department of Ophthalmology, University of Washington, Seattle, Washington, USA; 7 Roger and Angie Keralis Johnson Retina Center, Seattle, Washington, USA; 8 School of Computer Science and Mathematics, Kingston University, London, UK; 9 Department of Computer Science, Durham University, Durham, UK; 10 Division of Epidemiology and Clinical Applications, NEI/NIH, Bethesda, Maryland, USA; 11 Ophthalmic Research Consultants, Charlotte, North Carolina, USA; 12 Department of Pharmacology and Therapeutics, University of Liverpool, Liverpool, UK

**Keywords:** diabetic retinopathy, blindness, healthcare disparities, ethnic groups

## Abstract

**Introduction:**

The English Diabetic Eye Screening Programme (DESP) offers people living with diabetes (PLD) annual eye screening. We examined incidence and determinants of sight-threatening diabetic retinopathy (STDR) in a sociodemographically diverse multi-ethnic population.

**Research design and methods:**

North East London DESP cohort data (January 2012 to December 2021) with 137 591 PLD with no retinopathy, or non-STDR at baseline in one/both eyes, were used to calculate STDR incidence rates by sociodemographic factors, diabetes type, and duration. HR from Cox models examined associations with STDR.

**Results:**

There were 16 388 incident STDR cases over a median of 5.4 years (IQR 2.8–8.2; STDR rate 2.214, 95% CI 2.214 to 2.215 per 100 person-years). People with no retinopathy at baseline had a lower risk of sight-threatening diabetic retinopathy (STDR) compared with those with non-STDR in one eye (HR 3.03, 95% CI 2.91 to 3.15, p<0.001) and both eyes (HR 7.88, 95% CI 7.59 to 8.18, p<0.001). Black and South Asian individuals had higher STDR hazards than white individuals (HR 1.57, 95% CI 1.50 to 1.64 and HR 1.36, 95% CI 1.31 to 1.42, respectively). Additionally, every 5-year increase in age at inclusion was associated with an 8% reduction in STDR hazards (p<0.001).

**Conclusions:**

Ethnic disparities exist in a health system limited by capacity rather than patient economic circumstances. Diabetic retinopathy at first screen is a strong determinant of STDR development. By using basic demographic characteristics, screening programmes or clinical practices can stratify risk for sight-threatening diabetic retinopathy development.

**WHAT IS ALREADY KNOWN ON THIS TOPIC**

- There is little evidence about diabetic retinopathy and sight-threatening diabetic retinopathy (STDR) incidence rates in subgroups of multi-ethnic populations in the UK.
- Existing evidence shows non-white ethnic groups are more predisposed to diabetes and more prone to develop STDR when compared with white people.

**WHAT THIS STUDY ADDS**

- We provide STDR and any diabetic retinopathy incidence rates in a large sociodemographically diverse population.
- Baseline diabetic retinopathy severity and young age are strong predictors for STDR development.
- Non-white ethnic groups show greater incidence rates of STDR.

**HOW THIS STUDY MIGHT AFFECT RESEARCH, PRACTICE OR POLICY**

- Risk stratification provides clear progression rates to STDR based on routinely collected diabetic eye screening data from a single visit.
- These incidence rates for diabetic retinopathy and STDR are definitive, and of importance for power calculations for future research.

## Introduction

Diabetes is an increasing public health problem affecting 1 in 10 adults globally and a major cause of premature death and morbidity. The number of people with diabetes worldwide has almost quadrupled in the last two decades, from 151 million in the year 2000, to 537 million in 2021, and is projected to rise to 784 million in 2045, fueled by sizeable increases in low-income and middle-income countries.^1^

Diabetic retinopathy is a major complication of diabetes and a leading cause of incident sight impairment and blindness in the working age population.^2 3^ Early detection and intervention for sight-threatening diabetic retinopathy, once clinically defined criteria are met, can prevent blindness and indirectly reduce mortality.^4^ Healthcare costs are almost doubled for patients with diabetic retinopathy when compared with individuals without the disease. In the UK, the lifetime cost of dealing with a diabetic retinopathy case was estimated to be up to £237 000 per person in the working age group, from which half the costs accounted for lack of productivity due to visual loss.^5^ Since 2008, the UK offers nationwide annual diabetic eye screening (DES) to all people living with diabetes aged 12 years and older.^4^

Accurate identification of people with diabetes at high risk of sight-threatening complications remains a challenge.^5–7^ Established risk factors for diabetic retinopathy (namely, glycemic control, duration of disease, and systemic arterial hypertension) account for only a small proportion of the variation in risk.^8^ Demographic characteristics and social determinants of health are associated with diabetic retinopathy, however, there is little data from large-scale studies of sociodemographically diverse, multi-ethnic populations with standardised diabetic retinopathy grading, especially in the UK.

We report incidence rates (IR) of sight-threatening diabetic retinopathy, and examine sociodemographic associations of sight-threatening diabetic retinopathy, including ethnicity, age, sex, and deprivation in a large representative multi-ethnic population from the North East of London DES programme (NELDESP).

## Methods

The study population comprised 200 304 people with diabetes registered in the NELDESP who were offered screening appointments from January 3, 2012 to December 31, 2021.

### Setting

The North East of London is an ethnically diverse region with higher than national average levels of deprivation and mortality.^9^ The NELDESP is provided by the Homerton Healthcare NHS Foundation Trust, and serves people with diabetes living in inner-city areas with multi-ethnic populations. These are the boroughs of Newham, Redbridge, Tower Hamlets, and Waltham Forest, classified as the most ethnically diverse in London.^10^ The boroughs of Hackney, Havering, and the borough of Barking and Dagenham, also have a substantial multi-ethnic population. The NELDESP adheres to English NHS DESPDESP standards.^11^ All people with diabetes aged ≥12 years are identified through the electronic ‘General Practice to Diabetic Retinopathy Screening’ coding system, which automatically notifies DESPs about new diabetes diagnoses. All new eligible people are invited for screening within 3 months of notification. Software is used to generate invitations to attend for screening appointments. Over the course of 1 year, every person eligible for DES is offered multiple opportunities to attend.^12 13^

Briefly, a screening visit entails history taking by specialist staff, visual acuity assessment, and capture under pupil dilation of two 45° digital retinal images, centered on the fovea and optic nerve for each eye, respectively. Trained graders assess the images for presence and severity of diabetic retinopathy following a multilevel internally and externally quality-assured process.^11^ As per the UK National Screening Committee classification system (NSC-UK) for diabetic retinopathy,^14^ grades in order of increasing severity are: no retinopathy (R0), mild non-proliferative diabetic retinopathy (R1), severe non-proliferative diabetic retinopathy (R2), diabetic maculopathy (M1), and proliferative diabetic retinopathy (R3). Sight-threatening diabetic retinopathy (or referable diabetic retinopathy) comprises retinopathy grades greater than or equal to R2 and, for these, referral to hospital eye services for assessment/treatment is made.

### Data extraction and variables

We identified people registered in the NELDESP during the study period, calculated post code-derived index of multiple deprivation (IMD) rank scores for each episode and carried out an anonymised data extraction for all available appointments using structured query language searches. An anonymised database was created and stored within the Homerton Trust’s network for analysis.

We included data from people with non-sight-threatening diabetic retinopathy at baseline (defined as the first recorded screen), and with at least two complete screening visits (n=137 591; online supplemental figure 1).

10.1136/bmjdrc-2023-003683.supp1 Supplementary data

Routinely collected data from DES included age at first appointment (categorised as <45, 45 to <55, 55 to <65, and 65 years and older), sex, self-defined ethnicity (coded as per Office for National Statistics standards as: white, black, South Asian, Chinese, any other Asian, mixed, other, and unknown categories for the purpose of these analyses),^15^ type of diabetes (type 2, type 1, other, and unknown), self-defined duration of diabetes, baseline retinopathy severity (coded as diabetic retinopathy absent in both eyes (R0M0), non-sight-threatening diabetic retinopathy (R1M0) in one eye only, and non-sight-threatening diabetic retinopathy (R1M0) in both eyes),^6^ and IMD. The IMD combines and weights indicators of deprivation and is the nationally recognised measure of relative deprivation in England.^6 16^ IMD scores were split into quintiles (where first and fifth are the most and least deprived, respectively) in accordance with 2019 English indices of deprivation.^16^ Medication history, and metabolic data such as, haemoglobin A1c (HbA1c) or blood pressure measurements are not routinely collected in DES and were not available for analysis in this large cohort.

### Statistical analysis

The primary health outcome was progression to sight-threatening diabetic retinopathy (NSC-UK grades R2, R3, and/or M1) in at least one eye. We calculated IRs for any diabetic retinopathy and sight-threatening diabetic retinopathy. Our data are an example of panel data where, due to the intervals of the screening episodes, the date of diagnosis does not necessarily correspond to the date of change in retinopathy status. However, given a median (IQR) of 1.0 (0.9–1.1) years between appointments in the NELDESP, and since differences between models for interval and right censored data are expected to be small in studies where individuals attend at regular intervals,^17^ we undertook analyses using Cox proportional hazards (PH) model for right censored data (people who did not develop the outcome of interest were right censored at the time of last screening visit) to obtain HR for sight-threatening diabetic retinopathy adjusting for age, sex, baseline diabetic retinopathy, ethnicity, duration and type of diabetes, and IMD. Results from survival analysis for interval censored data are available as supplements. The proportionality assumption was assessed by graphical inspection of Schoenfeld residuals. As secondary analysis, we explored the associations for development of any diabetic retinopathy in people with no retinopathy at baseline with Cox regression.

Fully parametric accelerated failure time PH, and proportional odds models with different baseline distributions (Weibull, log-logistic, exponential, log-normal, and gamma) for interval censored data were fitted to obtain survival probabilities for sight-threatening diabetic retinopathy for people with different baseline characteristics. Parametric assumptions were tested graphically. The model with best fit defined by Akaike Information Criterion was the proportional odds model with baseline Weibull distribution. This model was used to create an online calculator to provide 10-year survival probabilities of individuals with different combinations of baseline characteristics (https://bit.ly/NEL-diabetic-eye-screening-risk-calculator).

Sensitivity analyses allowed for possible cumulative differences in duration of diabetes for people with baseline visits on the first two calendar years of our cohort by calculating IRs of sight-threatening diabetic retinopathy in the 2014–2021 cohort. All analyses were undertaken with R (V.4.2.2).^18^ The survival^17^ and icenReg^19^ packages were used for survival analyses.

## Results

A total of 137 591 people (73 840/137 591, 53.7% male) with a mean (SD) age of 56.8 (SD 14.8) years were included. There was a total of 16 388 incident sight-threatening diabetic retinopathy cases (82.9% M1, 13.2% R2, and 3.8% R3; online supplemental table 1 shows events in different ethnic groups by baseline diabetic retinopathy grade). Table 1 summarises our cohort baseline characteristics. Median (IQR) follow-up time was 5.4 (2.8–8.2) years. Among those that developed sight-threatening diabetic retinopathy, the median time to outcome was 4.0 (2.0–6.3) years overall, 5.2 (3.2–7.1) years for people with no retinopathy, 3.9 (2.1–6.1) years for people with retinopathy in one eye, and 2.7 (1.2–4.5) years for people with retinopathy in both eyes at baseline. Ethnicity codes were usable for 98.5% (135 487/137 591) of the population; 37% (50 907/137 591) were white and 42.3% (58 195/137 591) of the sample lived in areas with the two highest IMD quintiles of deprivation (1 and 2).

**Table 1:** Baseline population characteristics among those without sight-threatening diabetic retinopathy at baseline

Characteristic	Overall, n=137 591*	No retinopathy, n=107 701†	Retinopathy in one eye, n=17 570†	Retinopathy in both eyes, n=12 320†
Follow-up (years)	5.4 (2.8)	5.5 (2.8)	5.4 (2.9)	4.4 (2.9)
Age at baseline	56.8 (14.8)	56.5 (14.8)	58.0 (14.6)	57.1 (15.1)
Age category (years)				
<45	28 173 (20%)	22 552 (21%)	3124 (18%)	2497 (20%)
45 to <55	32 982 (24%)	26 038 (24%)	4115 (23%)	2829 (23%)
55 to <65	33 798 (25%)	26 373 (24%)	4425 (25%)	3000 (24%)
65 and over	42 638 (31%)	32 738 (30%)	5906 (34%)	3994 (32%)
Sex				
Female	63 751 (46%)	51 233 (48%)	7615 (43%)	4903 (40%)
Male	73 840 (54%)	56 468 (52%)	9955 (57%)	7417 (60%)
Type of diabetes				
2	128 270 (93%)	101 363 (94%)	16 301 (93%)	10 606 (86%)
1	5130 (3.7%)	3141 (2.9%)	736 (4.2%)	1253 (10%)
Other	251 (0.2%)	219 (0.2%)	15 (<0.1%)	17 (0.1%)
Missing	3940 (2.9%)	2978 (2.8%)	518 (2.9%)	444 (3.6%)
Ethnicity				
White	50 907 (37%)	39 663 (37%)	6423 (37%)	4821 (39%)
South Asian	47 994 (35%)	37 951 (35%)	5992 (34%)	4051 (33%)
Black	22 095 (16%)	17 303 (16%)	2907 (17%)	1885 (15%)
Any other Asian	7741 (5.6%)	6013 (5.6%)	1039 (5.9%)	689 (5.6%)
Other	4051 (2.9%)	3097 (2.9%)	594 (3.4%)	360 (2.9%)
Mixed	1744 (1.3%)	1373 (1.3%)	206 (1.2%)	165 (1.3%)
Chinese	955 (0.7%)	745 (0.7%)	119 (0.7%)	91 (0.7%)
Unknown	2104 (1.5%)	1556 (1.4%)	290 (1.7%)	258 (2.1%)
Duration of diabetes	4.7 (6.4)	3.9 (5.4)	6.2 (7.2)	10.1 (9.2)
Index of multiple deprivation				
1	14 598 (11%)	11 537 (11%)	1792 (10%)	1269 (10%)
2	43 597 (32%)	34 155 (32%)	5527 (31%)	3915 (32%)
3	39 756 (29%)	31 167 (29%)	5064 (29%)	3525 (29%)
4	25 906 (19%)	20 114 (19%)	3408 (19%)	2384 (19%)
5	13 541 (9.8%)	10 600 (9.8%)	1746 (9.9%)	1195 (9.7%)
Missing	193 (0.1%)	128 (0.1%)	33 (0.2%)	32 (0.3%)

*Mean (SD) for continuous variables, n (column %) for categorical variables.

†No retinopathy (R0M0); retinopathy in one eye (R1M0 in one eye); retinopathy in both eyes (R1M0 in both eyes).

### Prevalence and incidence of diabetic retinopathy and sight-threatening diabetic retinopathy

At first recorded visit, the point prevalence of any retinopathy and sight-threatening diabetic retinopathy was 27.5% (48 628/176 767), and 8.1% (14 231/176 767), respectively. The cumulative IR (CIR) of sight-threatening diabetic retinopathy was 2.21 (95% CI 2.21 to 2.21) per 100 person-years. Table 2 shows CIRs of sight-threatening diabetic retinopathy per 100 person-years by follow-up. Baseline retinopathy severity showed a strong relationship with sight-threatening diabetic retinopathy rates. Progression to sight-threatening diabetic retinopathy with advancing yearly intervals showed an overall monotonic increase in rates, which was more pronounced in younger age groups when compared with the consistently lower rates in older age groups (online supplemental table 2). Sensitivity analyses excluding the earliest 2 years of the study period did not materially alter sight-threatening diabetic retinopathy CIR (online supplemental table 3).

**Table 2:** Cumulative incidence rates per 100 person-years for development of sight-threatening DR in at least one eye by length of follow-up with 95% CIs in parenthesis

Characteristic	Year 1	Year 2	Year 3	Year 4	Year 5	Year 10
Overall	0.86 (0.82 to 0.90)	1.48 (1.43 to 1.54)	1.66 (1.61 to 1.72)	1.78 (1.73 to 1.84)	1.89 (1.83 to 1.95)	2.21 (2.15 to 2.28)
Age group (years)						
<45	0.90 (0.81 to 0.99)	1.59 (1.47 to 1.72)	1.82 (1.69 to 1.95)	2.02 (1.88 to 2.15)	2.20 (2.05 to 2.34)	2.84 (2.68 to 3.01)
45 to <55	0.86 (0.78 to 0.95)	1.54 (1.43 to 1.66)	1.76 (1.64 to 1.88)	1.86 (1.73 to 1.98)	2.01 (1.88 to 2.13)	2.42 (2.28 to 2.55)
55 to <65	0.85 (0.77 to 0.93)	1.42 (1.31 to 1.52)	1.57 (1.46 to 1.69)	1.68 (1.56 to 1.79)	1.73 (1.61 to 1.84)	1.96 (1.84 to 2.09)
65 and over	0.84 (0.77 to 0.91)	1.41 (1.32 to 1.51)	1.55 (1.46 to 1.65)	1.66 (1.56 to 1.76)	1.72 (1.62 to 1.83)	1.87 (1.76 to 1.98)
Sex						
Female	0.78 (0.72 to 0.84)	1.36 (1.28 to 1.43)	1.55 (1.47 to 1.63)	1.68 (1.59 to 1.76)	1.77 (1.68 to 1.85)	2.08 (1.98 to 2.17)
Male	0.93 (0.87 to 0.99)	1.59 (1.51 to 1.67)	1.76 (1.68 to 1.84)	1.88 (1.80 to 1.96)	1.99 (1.91 to 2.08)	2.34 (2.25 to 2.43)
Ethnicity						
White	0.76 (0.69 to 0.82)	1.25 (1.17 to 1.33)	1.40 (1.31 to 1.49)	1.50 (1.42 to 1.59)	1.56 (1.47 to 1.65)	1.77 (1.68 to 1.87)
South Asian	0.85 (0.78 to 0.92)	1.53 (1.44 to 1.62)	1.71 (1.61 to 1.80)	1.85 (1.75 to 1.95)	1.99 (1.88 to 2.09)	2.44 (2.32 to 2.55)
Black	1.08 (0.97 to 1.19)	1.87 (1.72 to 2.02)	2.09 (1.93 to 2.25)	2.19 (2.03 to 2.36)	2.32 (2.15 to 2.49)	2.68 (2.50 to 2.86)
Any other Asian	0.84 (0.67 to 1.01)	1.52 (1.29 to 1.75)	1.68 (1.44 to 1.92)	1.86 (1.61 to 2.12)	2.02 (1.76 to 2.28)	2.24 (1.97 to 2.52)
Other	1.07 (0.80 to 1.34)	1.42 (1.11 to 1.72)	1.70 (1.37 to 2.04)	1.79 (1.45 to 2.14)	1.90 (1.55 to 2.26)	2.33 (1.94 to 2.71)
Mixed	0.75 (0.41 to 1.09)	1.33 (0.88 to 1.78)	1.70 (1.19 to 2.21)	1.94 (1.40 to 2.49)	2.14 (1.57 to 2.71)	2.41 (1.81 to 3.02)
Chinese	1.16 (0.59 to 1.73)	1.62 (0.95 to 2.29)	1.81 (1.10 to 2.52)	1.73 (1.04 to 2.43)	1.69 (1.00 to 2.38)	1.65 (0.97 to 2.33)
Unknown	0.91 (0.57 to 1.26)	2.09 (1.57 to 2.60)	2.51 (1.95 to 3.07)	2.59 (2.02 to 3.16)	2.64 (2.07 to 3.22)	2.61 (2.03 to 3.18)
Baseline DR grade						
No retinopathy	0.21 (0.19 to 0.23)	0.44 (0.41 to 0.48)	0.56 (0.52 to 0.59)	0.66 (0.62 to 0.70)	0.79 (0.75 to 0.84)	1.20 (1.15 to 1.26)
Retinopathy in one eye	1.23 (1.10 to 1.37)	2.37 (2.18 to 2.56)	2.78 (2.58 to 2.99)	3.16 (2.94 to 3.37)	3.33 (3.11 to 3.55)	3.80 (3.56 to 4.03)
Retinopathy in both eyes	6.04 (5.69 to 6.39)	9.81 (9.37 to 10.26)	10.61 (10.15 to 11.06)	10.78 (10.32 to 11.24)	10.77 (10.31 to 11.23)	10.43 (9.98 to 10.88)

DR, diabetic retinopathy.

Cumulative incidence of any retinopathy and of sight-threatening diabetic retinopathy for participants with no retinopathy at baseline was 35.3% (38 059/107 701) and 6.6% (7116/107 701), respectively. The CIR (95% CI) of any retinopathy was 7.95 (7.95 to 7.95) per 100 person-years. The highest CIR for any retinopathy was observed in the other and South Asian ethnic groups (8.38 (95% CI 8.36 to 8.39) and 8.12 (95% CI 8.12 to 8.12) per 100 person-years, respectively). The highest CIR for sight-threatening diabetic retinopathy was observed in people of black and South Asian ethnicities (2.67 (95% CI 2.67 to 2.67) and 2.44 (95% CI 2.44 to 2.44) per 100 person-years, respectively). People of any other Asian ethnicity had the lowest IR for any diabetic retinopathy (7.48 (95% CI 7.47 to 7.48) per 100 person-years). People of Chinese ethnicity had the lowest CIR for sight-threatening diabetic retinopathy (1.65 (95% CI 1.63 to 1.67) per 100 person-years, respectively (online supplemental table 4)). CIR of any retinopathy per 100 person-years by follow-up is shown in online supplemental table 5.

### Survival probabilities and sociodemographic associations with sight-threatening diabetic retinopathy

**Figure 1:**
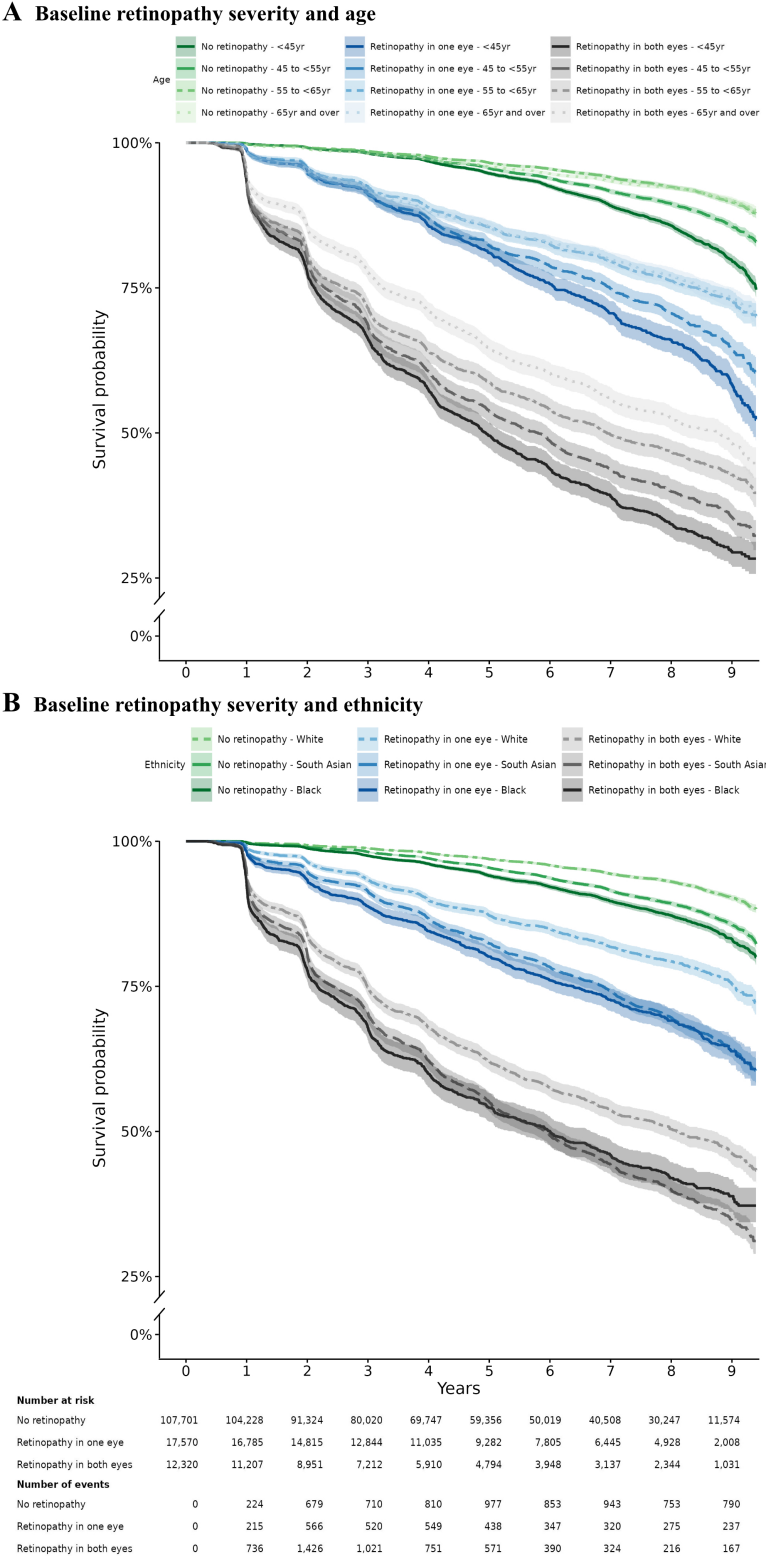
Survival plots for development of sight-threatening diabetic retinopathy (STDR). Stratified by non-STDR at baseline (no retinopathy, non-STDR in one eye, non-STDR in both eyes) and age (A), and non-STDR at baseline and ethnicity (B). Shaded areas represent 95% CI.

Survival probabilities for sight-threatening diabetic retinopathy development stratified by retinopathy severity at baseline and age groups were consistently lower in younger people with non-sight-threatening diabetic retinopathy in both eyes at baseline (figure 1A). By year 5, 50.5% (95% CI 48.2 to 52.6) of those in the youngest age group (<45 years) with non-sight-threatening diabetic retinopathy at baseline in both eyes progressed to sight-threatening diabetic retinopathy compared with 19.0% (95% CI 17.4 to 20.6) in the same age group but with non-sight-threatening diabetic retinopathy in one eye only (figure 1A). Survival curves stratified by retinopathy severity at baseline and the three major ethnic groups consistently showed lower survival probabilities for non-white ethnicities (figure 1B). At year 5, 46.0% (95% CI 43.4 to 48.3) and 45.0% (95% CI 43.3 to 46.6) of black and South Asian people with non-sight-threatening diabetic retinopathy in both eyes progressed to sight-threatening diabetic retinopathy, respectively. In contrast, at the same timepoint, 37.9% (95% CI 36.3 to 39.4) of white people with non-sight-threatening diabetic retinopathy in both eyes progressed to sight-threatening diabetic retinopathy. A Kaplan-Meier plot for interval censored data showed comparable findings (online supplemental figure 2).

Table 3 summarises mutually adjusted HRs from our multivariable Cox model. Age categories showed a strong graded inverse association with hazards of sight-threatening diabetic retinopathy (p for linear trend <0.0001). Males showed a 4% increase in sight-threatening diabetic retinopathy hazards when compared with females (p=0.011). When compared with people with no retinopathy at baseline, people with non-sight-threatening diabetic retinopathy in one eye had a threefold increase in sight-threatening diabetic retinopathy hazards, whereas people with non-sight-threatening diabetic retinopathy in both eyes at baseline showed an eightfold increase in sight-threatening diabetic retinopathy hazards.

**Table 3:** Hazard ratios (HR) mutually adjusted for all factors shown

Characteristic	HR (95% CI)*	P value
Age (per 5-year increase)†	0.92 (0.92 to 0.93)	**1.0e-133**
Age category (years)		
<45	1.00	
45 to <55	0.79 (0.75 to 0.82)	**1.5e-26**
55 to <65	0.60 (0.58 to 0.63)	**1.1e-96**
65 and over	0.57 (0.54 to 0.59)	**1.4e-115**
Sex		
Female	1.00	
Male	1.04 (1.01 to 1.07)	**0.011**
Baseline DR grade‡		
No retinopathy	1.00	
Retinopathy in one eye	3.03 (2.91 to 3.15)	**4.6e-626**
Retinopathy in both eyes	7.88 (7.59 to 8.18)	**2.5e-2521**
Ethnicity		
White	1.00	
South Asian	1.36 (1.31 to 1.42)	**1.0e-52**
Black	1.57 (1.50 to 1.64)	**2.6e-81**
Any other Asian	1.25 (1.16 to 1.34)	**4.0e-10**
Other	1.29 (1.18 to 1.42)	**4.7e-08**
Mixed	1.39 (1.20 to 1.60)	**5.7e-06**
Chinese	0.98 (0.79 to 1.21)	0.831
Unknown	1.71 (1.41 to 2.07)	**4.8e-08**
Duration of diabetes (per 5-year increase)	1.14 (1.13 to 1.15)	**8.3e-138**
Type of diabetes		
2	1.00	
1	1.03 (0.97 to 1.11)	0.337
Other	0.73 (0.44 to 1.22)	0.231
Missing	1.34 (1.20 to 1.51)	**6.3e-07**
Deprivation (IMD quintiles)		
1	1.00	
2	1.04 (0.99 to 1.10)	0.141
3	1.03 (0.98 to 1.09)	0.281
4	0.98 (0.93 to 1.04)	0.592
5	0.93 (0.87 to 1.00)	**0.041**
Missing	1.68 (0.75 to 3.75)	0.208

HR >1 imply greater hazards for sight-threatening DR. Bold p values show statistically significant results.

*Mutually adjusted HR from Cox model adjusting for age, sex, baseline DR severity, ethnicity, duration and type of diabetes and IMD.

†Models with age as continuous and categorical variable were fitted separately. Estimates are included in this table to show non-linear associations.

‡No retinopathy (R0M0 in both eyes); retinopathy in one eye (R1M0 in one eye); retinopathy in both eyes (R1M0 in both eyes).

DR, diabetic retinopathy; IMD, index of multiple deprivation.

When compared with the white ethnic group, non-white ethnic groups showed increased sight-threatening diabetic retinopathy hazards. The biggest effect sizes were observed in people of black (HR 1.57; 95% CI 1.50 to 1.64), mixed (HR 1.39; 95% CI 1.20 to 1.60), and South Asian (HR 1.36; 95% CI 1.31 to 1.42) ethnicity. The Chinese ethnic group did not show differences for sight-threatening diabetic retinopathy when compared with white individuals. Formal tests for interaction between ethnicity with IMD were not statistically significant (p=0.21).

Every 5-year increase in duration of diabetes conferred a 14% increase in hazards of sight-threatening diabetic retinopathy. When compared with the highest deprivation quintile, only the least deprived quintile of IMD showed a 7% reduction in hazards of sight-threatening diabetic retinopathy (p=0.041). However, IMD was no longer formally statistically significant using interval censoring (online supplemental table 6). Type of diabetes did not appear to be associated with sight-threatening diabetic retinopathy in the adjusted regression model.

Associations for development of any diabetic retinopathy in people with no retinopathy at baseline (R0M0, n=107 701) are shown in online supplemental table 7. When compared with type 2 diabetes, people with type 1 diabetes showed a strong association with the larger effect size for any retinopathy (HR 1.36, 95% CI 1.28 to 1.44), and every 5-year rise in duration of diabetes was associated with 18% increase in diabetic retinopathy hazards. Every 5-year increase in age conferred a 1% decrease in diabetic retinopathy hazards, males had a 6% increase in diabetic retinopathy hazards when compared with females, and ethnic differences were considerably less pronounced and only statistically significantly different from whites, for South Asian (HR 1.03, 95% CI 1.00 to 1.06), other (HR 1.09, 95% CI 1.02 to 1.16), and unknown (HR 1.36, 95% CI 1.21 to 1.54) ethnic groups. No associations were observed for IMD and any diabetic retinopathy.

## Conclusions

Our study provides definitive IRs for sight-threatening diabetic retinopathy by demographic characteristics routinely collected in DES. There is a strong relationship in CIR increases with retinopathy severity at baseline, and a monotonic increase in rates with advancing yearly intervals, more pronounced in younger people (table 2 and online supplemental table 2). Presence of retinopathy in both eyes, age, and ethnicity are strong determinants for incident sight-threatening diabetic retinopathy. We have defined and calculated sociodemographic associations with sight-threatening diabetic retinopathy, and we provide a web calculator (https://bit.ly/NEL-diabetic-eye-screening-risk-calculator) to estimate disease trajectories of individuals with different sociodemographic profiles. Young, male, non-white ethnic groups with longer duration of diabetes show higher sight-threatening diabetic retinopathy hazards when compared with older, female, white groups, and those with shorter duration of diabetes.

Diabetic retinopathy status at baseline was the most important predictor for sight-threatening diabetic retinopathy referral to hospital eye services. When compared with people with no retinopathy at baseline, the threefold and almost eightfold increase in hazards of sight-threatening diabetic retinopathy for non-sight-threatening diabetic retinopathy in one eye and non-sight-threatening diabetic retinopathy in both eyes, respectively, is consistent with the reported HRs in the literature.^5 6^ The absence of diabetic retinopathy and the presence of non-sight-threatening diabetic retinopathy in one or both eyes provides valuable information for simple risk stratification in groups of people that were previously considered of low homogeneous risk.^6^

Younger age groups have been reported to have higher incidence of sight-threatening diabetic retinopathy^20–24^ and a recent analysis from observed data has shown that young people are at increased risk of experiencing significant delays in diagnosis of sight-threatening diabetic retinopathy if biennial screening intervals were to be implemented.^25^ Derived from our analysis, we provide evidence of the presence of a different disease trajectory with increased risk of sight-threatening diabetic retinopathy in younger people (HR per 5-year rise in age 0.92, p<0.001). The causes are likely multiple, and result from a complex interplay of different factors that could be partially explained by higher levels of non-attendance to DES,^12 13^ and by suboptimal control of diabetes and major modifiable risk factors^26 27^ in young people. Individuals younger than 45 years of age are at a critical stage of their work, or career development, and the lifetime burden and health costs of sight-threatening complications in this population is of considerable public health importance.

Males are at higher risk of sight-threatening diabetic retinopathy development than females. Similar effect sizes to what is reported in our study are available.^12 21 24^ An electronic medical record (EMR)-based study analysing the development of sight-threatening diabetic retinopathy in people with diabetes in the UK found an HR of 1.22 (95% CI 1.01 to 1.47) and 1.15 (95% CI 1.06 to 1.26) for males newly diagnosed, and males with known diagnosis of diabetes when compared with females, respectively.^21^ Similarly, Mathur *et al* reported an increased relative risk of diabetic retinopathy (HR 1.08, 95% CI 1.05 to 1.09), and severe diabetic retinopathy (HR 1.25, 95% CI 1.12 to 1.39), in males when compared with females.^22^ Lawrenson *et al* found a 23% increase in odds (95% CI 1.15 to 1.35) of sight-threatening diabetic retinopathy in males when compared with females in a 15-month limited follow-up DES study.^12^ Non-attendance to DES,^12 13^ and hormonal differences^28^ are possible underpinning factors, but the evidence remains contradictory.

South Asian and black ethnic groups have been reported to have higher prevalence of diabetes,^22 29 30^ and are more likely to develop both sight-threatening diabetic retinopathy^30^ and visual impairment^31^ than white people. Sivaprasad *et al* 30 reported, in a predominantly white (66%) population, an 82% (HR 1.82, 95% CI 1.61 to 2.06) and 99% (HR 1.99, 95% CI 1.81 to 2.18) increase in risk of sight-threatening diabetic retinopathy in South Asian and black ethnic groups when compared with white people, respectively. Mathur *et al* 22 showed a 25% (HR 1.25, 95% CI 1.00 to 1.56) increase in risk of severe retinopathy (defined as advanced, proliferative, or laser-treated diabetic retinopathy) in South Asian patients when compared with white patients, however, a third of the ethnicity data were missing. Scanlon *et al* 5 showed a 55%, 58%, and 24% increase in hazards of sight-threatening diabetic retinopathy in African, Caribbean, and other ethnic groups when compared with white people in a small (n=1 223) dataset from South London. More recently, an EMR-based study with over 98% with usable ethnic coding, identified increased risk of sight-threatening diabetic retinopathy in African (HR 1.36, 95% CI 1.02 to 1.83), Indian (HR 1.38, 95% CI 1.17 to 1.63), Pakistani (HR 1.28, 95% CI 1.04 to 1.55), Bangladeshi (HR 1.36, 95% CI 1.19 to 1.54), Caribbean (HR 1.22, 95% CI 1.03 to 1.43), and other (HR 1.25, 95% CI 1.06 to 1.47) ethnicities with a new diagnosis of diabetes when compared with white individuals.^21^ And Mangelis *et al* 32 identified a 39% increase in hazards of sight-threatening diabetic retinopathy (p=0.009) in African Caribbeans with type 1 diabetes when compared with non-African Caribbean people. These results stress the need to address health inequalities across ethnic groups to improve prevention of sight-threatening complications.

Our study has several strengths. First, a large sample size providing considerable statistical power to detect associations (or absence of association) with sight-threatening diabetic retinopathy stratified by age, sex, ethnicity, retinopathy severity at baseline, and deprivation. Second, IRs provide an important source of reference to inform power calculations for future clinical trials. Third, there is high quality in ethnicity recording with usability of 98.5%. Fourth, the prevalence of any diabetic retinopathy falls in line with previous reports (27.5% prevalence overall, 49.1% and 26.4% in people with type 1 and type 2 diabetes, respectively) and is representative of the UK.^5 22 33^ Fifth, retinopathy classification was performed by trained assessors following a multilevel internally and externally quality-assured grading protocol that meets UK national recommendations.^11^

The limitations of our study are as follows. First, HbA1c, blood pressure, blood lipids, medication history, or body mass index were not available for analysis. However, estimates of our Cox model are in alignment with reports from a previous EMR-based study which controlled for the above-mentioned variables.^21^ Second, we cannot exclude human errors in grading of retinal fundus images despite the well-established grading protocol, but it is expected that, based on hospital eye service outcomes, findings of the study would not substantially differ.

Leveraging an unprecedented multi-ethnic and sociodemographically diverse population undergoing DES, our study delivers a contemporary analysis of sight-threatening diabetic retinopathy incidence and associated factors. IRs provided in our analysis are valuable for future research requiring estimates of transition probabilities or sample size. Our survival analysis revealed significant associations based on simple sociodemographic variables available in routine DES which offer significant information for risk stratification among people with diabetes. Understanding IR and associations of sight-threatening diabetic retinopathy in cohorts such as ours, illuminates paths for future research, identifies areas to optimise service planning, and equity in eye care. Further work to devise accurate prediction models and assess the potential contribution of clinical/metabolic, imaging, and imaging-derived data to risk prediction are essential next steps.

## Data Availability

Data are available on reasonable request. The North East London Diabetic Eye Screening Programme data are not publicly available because of restrictions on data sharing. A fully anonymized dataset is available from the Programme on reasonable request.
