# Supplementary material for: Ethnic disparities in progression rates for sight-threatening diabetic retinopathy in diabetic eye screening: a population-based retrospective cohort study
Source: BMJ Open Diabetes Res Care. 2023 Nov 10;11(6):e003683. doi: 10.1136/bmjdrc-2023-003683 (PMC10649497; doi:10.1136/bmjdrc-2023-003683)
Supplement: Supplementary data [file bmjdrc-2023-003683supp001.pdf]

Supplementary material

Table of Contents

Supplementary figure 1. Diagram of exclusions. 2

Supplementary figure 2. Survival plot for development of referable diabetic retinopathy fitted with interval censoring and the Turnbull estimator stratified by baseline diabetic retinopathy grade and age. . 3

Supplementary table 1. Sight-threatening diabetic retinopathy events in ethnic groups by baseline diabetic retinopathy grade at baseline. 4

Supplementary table 2. Incidence rate of sight-threatening-diabetic-retinopathy per 100 person-years by follow-up period (95% confidence intervals). 5

Supplementary table 3. Sensitivity analysis using incidence rate of referable diabetic retinopathy per 100 person-years by follow-up period defined by 95% confidence intervals. Excluding people from the earliest 2 years of study period (see table 3 of our manuscript for comparison). 6

Supplementary table 4. Incidence rates of any diabetic retinopathy (people with R0M0 at first screen, n = 107,701) and sight-threatening diabetic retinopathy (people with R0M0 and R1M0 at first screen, n = 137,591) per 100 person-years by ethnic group. 7

Supplementary table 5. Cumulative incidence rate of any diabetic retinopathy per 100 person-years by follow-up (95% confidence intervals). 8

Supplementary table 6. Semi-parametric multivariable Cox proportional hazards model for interval censored data. Hazard ratios (HR) are mutually adjusted for all factors shown in the table. HR greater than 1 imply greater hazards for vision-threatening diabetic retinopathy (DR). Bold p-values show statistically significant results. 9

Supplementary table 7. Multivariable Cox proportional hazards model for right censored data with development of any diabetic retinopathy (DR) as outcome of interest. Hazard ratios (HR) are mutually adjusted for all factors shown in the table. HR greater than 1 imply greater hazards for DR. Bold p-values show statistically significant results. 10

Supplementary figure 1. Diagram of exclusions.

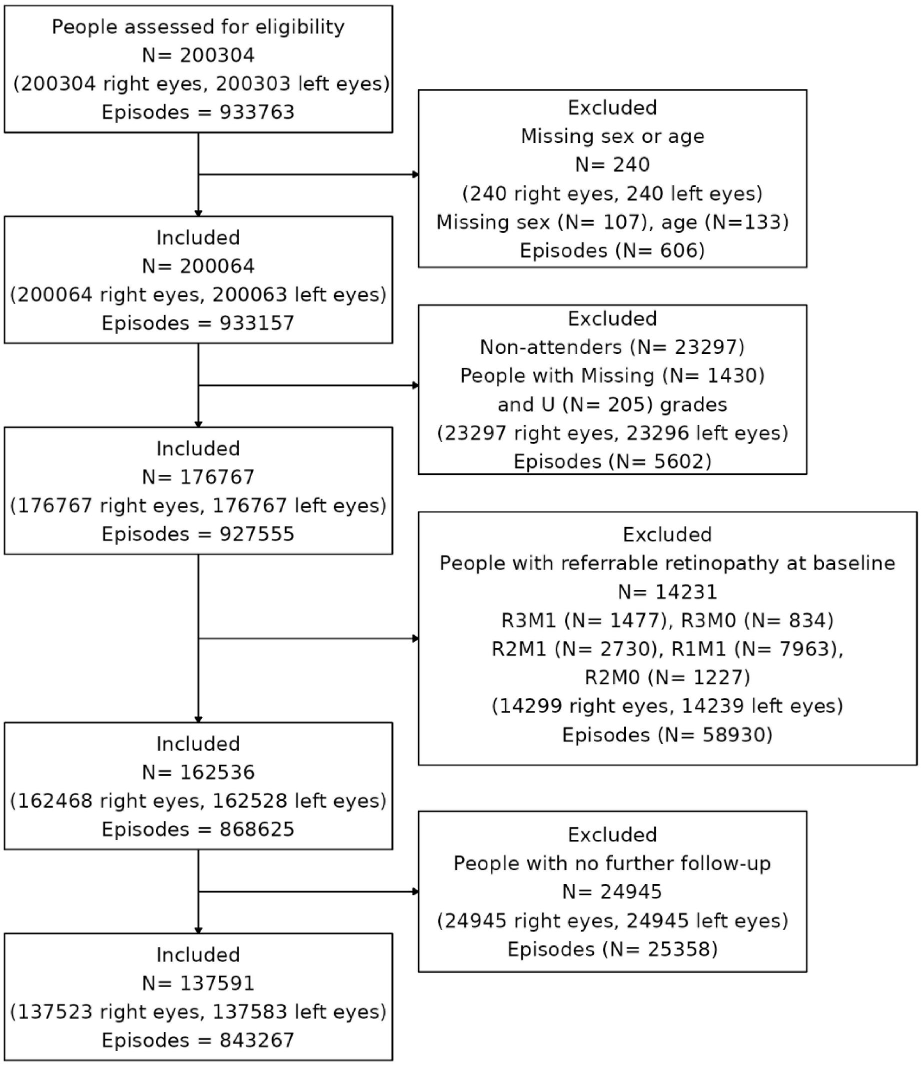

Supplementary figure 2. Survival plot for development of referable diabetic retinopathy fitted with interval censoring and the Turnbull estimator stratified by baseline diabetic retinopathy grade and age.

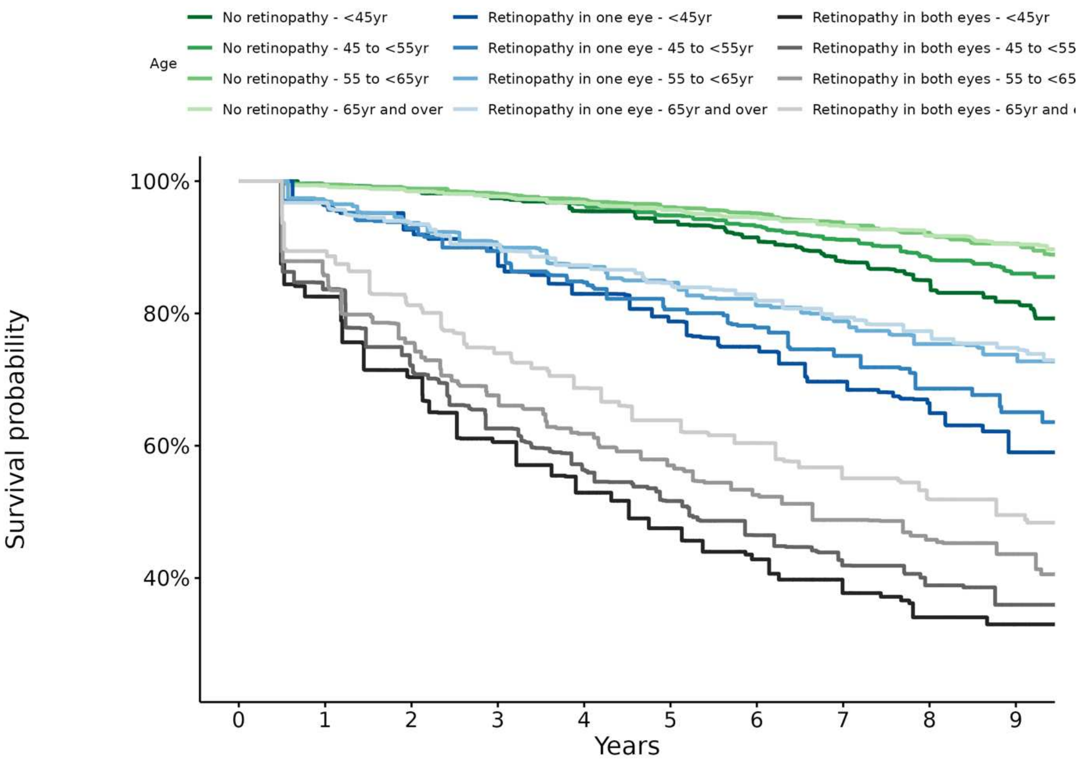

Number at risk

	0	1	2	3	4	5	6	7	8	9
No retinopathy	107701	104228	91324	80020	69747	59356	50019	40508	30247	11574
Retinopathy in one eye	17570	16785	14815	12844	11035	9282	7805	6445	4928	2008
Retinopathy in both eyes	12320	11207	8951	7212	5910	4794	3948	3137	2344	1031

Cumulative number of events

	0	1	2	3	4	5	6	7	8	9
No retinopathy	0	224	903	1613	2423	3400	4253	5196	5949	6739
Retinopathy in one eye	0	215	781	1301	1850	2288	2635	2955	3230	3467
Retinopathy in both eyes	0	736	2162	3183	3934	4505	4895	5219	5435	5602

Supplementary table 1. Sight-threatening diabetic retinopathy events in ethnic groups by baseline diabetic retinopathy grade at baseline.

**Characteristic**	**Overall**, N = 16,388	**No retinopathy**, N = 7,116	**Retinopathy in one eye**, N = 3,583	**Retinopathy in both eyes**, N = 5,689
**Ethnicity**				
White	4,782 (29%)	1,854 (26%)	992 (28%)	1,936 (34%)
South Asian	6,532 (40%)	2,942 (41%)	1,463 (41%)	2,127 (37%)
Black	3,186 (19%)	1,542 (22%)	716 (20%)	928 (16%)
Any other Asian	990 (6.0%)	394 (5.5%)	239 (6.7%)	357 (6.3%)
Other	503 (3.1%)	231 (3.2%)	106 (3.0%)	166 (2.9%)
Mixed	201 (1.2%)	88 (1.2%)	34 (0.9%)	79 (1.4%)
Chinese	85 (0.5%)	29 (0.4%)	15 (0.4%)	41 (0.7%)
Unknown	109 (0.7%)	36 (0.5%)	18 (0.5%)	55 (1.0%)

n (column %)

Supplementary table 2. Incidence rate of sight-threatening-diabetic-retinopathy per 100 person-years by follow-up period (95% confidence intervals).

**Characteristic**	**0-1yr**	**1-2yr**	**2-3yr**	**3-4yr**	**4-5yr**	**5-10yr**
**Overall**	0.86 (0.82 - 0.90)	2.18 (2.11 - 2.24)	2.09 (2.03 - 2.16)	2.26 (2.19 - 2.34)	2.48 (2.39 - 2.57)	3.10 (2.99 - 3.20)
**Age groups**						
Less than 45 years	0.90 (0.81 - 0.99)	2.38 (2.23 - 2.53)	2.39 (2.22 - 2.56)	2.82 (2.62 - 3.01)	3.32 (3.09 - 3.55)	4.89 (4.58 - 5.20)
45 to <55 years	0.86 (0.78 - 0.95)	2.29 (2.15 - 2.43)	2.26 (2.12 - 2.41)	2.25 (2.09 - 2.40)	2.85 (2.66 - 3.03)	3.49 (3.26 - 3.71)
55 to <65 years	0.85 (0.77 - 0.93)	2.04 (1.91 - 2.17)	1.94 (1.81 - 2.08)	2.07 (1.92 - 2.22)	2.00 (1.85 - 2.15)	2.54 (2.35 - 2.72)
65 years and over	0.84 (0.77 - 0.91)	2.06 (1.94 - 2.18)	1.90 (1.78 - 2.01)	2.09 (1.96 - 2.23)	2.08 (1.93 - 2.22)	2.28 (2.11 - 2.45)
**Sex**						
Female	0.78 (0.72 - 0.84)	2.00 (1.91 - 2.09)	2.02 (1.92 - 2.12)	2.15 (2.04 - 2.26)	2.29 (2.17 - 2.41)	2.89 (2.74 - 3.04)
Male	0.93 (0.87 - 0.99)	2.33 (2.24 - 2.42)	2.16 (2.06 - 2.26)	2.36 (2.25 - 2.47)	2.65 (2.53 - 2.77)	3.29 (3.14 - 3.44)
**Ethnicity**						
White	0.76 (0.69 - 0.82)	1.80 (1.70 - 1.90)	1.76 (1.66 - 1.87)	1.92 (1.80 - 2.04)	1.87 (1.75 - 2.00)	2.37 (2.21 - 2.52)
South Asian	0.85 (0.78 - 0.92)	2.28 (2.16 - 2.39)	2.12 (2.00 - 2.23)	2.40 (2.27 - 2.53)	2.74 (2.59 - 2.89)	3.62 (3.43 - 3.80)
Black	1.08 (0.97 - 1.19)	2.75 (2.56 - 2.93)	2.63 (2.44 - 2.82)	2.59 (2.38 - 2.80)	3.05 (2.81 - 3.29)	3.65 (3.36 - 3.93)
Any other Asian	0.84 (0.67 - 1.01)	2.26 (1.98 - 2.54)	2.04 (1.76 - 2.33)	2.57 (2.23 - 2.91)	2.87 (2.49 - 3.25)	2.80 (2.40 - 3.21)
Other	1.07 (0.80 - 1.34)	1.81 (1.46 - 2.16)	2.39 (1.96 - 2.83)	2.16 (1.72 - 2.61)	2.53 (2.02 - 3.05)	3.46 (2.81 - 4.11)
Mixed	0.75 (0.41 - 1.09)	1.98 (1.42 - 2.54)	2.65 (1.95 - 3.36)	3.00 (2.17 - 3.83)	3.44 (2.46 - 4.42)	3.31 (2.25 - 4.37)
Chinese	1.16 (0.59 - 1.73)	2.13 (1.35 - 2.92)	2.29 (1.42 - 3.16)	1.41 (0.67 - 2.15)	1.44 (0.64 - 2.24)	1.56 (0.66 - 2.46)
Unknown	0.91 (0.57 - 1.26)	4.18 (3.36 - 5.00)	5.20 (3.88 - 6.51)	3.77 (2.03 - 5.51)	4.06 (1.73 - 6.39)	2.02 (-0.10 - 4.15)
**Baseline DR grade**						
No retinopathy	0.21 (0.19 - 0.23)	0.70 (0.66 - 0.74)	0.83 (0.78 - 0.88)	1.08 (1.02 - 1.14)	1.51 (1.43 - 1.59)	2.30 (2.20 - 2.40)
Retinopathy in one eye	1.23 (1.10 - 1.37)	3.63 (3.40 - 3.87)	3.79 (3.53 - 4.05)	4.61 (4.31 - 4.92)	4.33 (4.01 - 4.65)	5.05 (4.68 - 5.42)
Retinopathy in both eyes	6.04 (5.69 - 6.39)	14.48 (13.94 - 15.03)	12.79 (12.21 - 13.37)	11.57 (10.95 - 12.19)	10.75 (10.08 - 11.41)	9.30 (8.61 - 9.99)

Olvera-Barrios A, *et al. BMJ Open Diab Res Care* 2023; 11:e003683. doi: 10.1136/bmjdrc-2023-003683

Supplementary table 3. Sensitivity analysis using incidence rate of referable diabetic retinopathy per 100 person-years by follow-up period defined by 95% confidence intervals. Excluding people from the earliest 2 years of study period (see table 3 of our manuscript for comparison).

Characteristic	0-1yr	1-2yr	2-3yr	3-4yr	4-5yr	5-8yr
**Overall**	0.65 (0.60 - 0.71)	1.65 (1.56 - 1.74)	1.56 (1.46 - 1.66)	1.69 (1.57 - 1.81)	2.37 (2.21 - 2.54)	2.99 (2.76 - 3.22)
**Age groups**						
Less than 45 years	0.88 (0.76 - 1.01)	1.98 (1.79 - 2.17)	1.92 (1.71 - 2.13)	2.31 (2.04 - 2.57)	3.34 (2.97 - 3.72)	3.87 (3.36 - 4.38)
45 to <55 years	0.57 (0.47 - 0.67)	1.58 (1.41 - 1.75)	1.67 (1.47 - 1.87)	1.48 (1.27 - 1.69)	2.42 (2.11 - 2.73)	2.94 (2.52 - 3.37)
55 to <65 years	0.54 (0.44 - 0.65)	1.36 (1.19 - 1.53)	1.28 (1.09 - 1.46)	1.59 (1.36 - 1.83)	1.90 (1.60 - 2.19)	2.39 (1.97 - 2.80)
65 years and over	0.59 (0.48 - 0.70)	1.64 (1.45 - 1.83)	1.29 (1.10 - 1.49)	1.29 (1.07 - 1.52)	1.64 (1.34 - 1.94)	2.64 (2.17 - 3.12)
**Sex**						
Female	0.53 (0.46 - 0.60)	1.37 (1.25 - 1.49)	1.30 (1.17 - 1.43)	1.42 (1.26 - 1.59)	2.06 (1.83 - 2.28)	2.66 (2.34 - 2.98)
Male	0.76 (0.68 - 0.84)	1.89 (1.76 - 2.02)	1.78 (1.64 - 1.93)	1.91 (1.74 - 2.09)	2.64 (2.41 - 2.88)	3.27 (2.94 - 3.60)
**Ethnicity**						
White	0.51 (0.42 - 0.59)	1.40 (1.25 - 1.54)	1.40 (1.24 - 1.56)	1.61 (1.41 - 1.81)	1.88 (1.62 - 2.14)	2.21 (1.86 - 2.56)
South Asian	0.73 (0.63 - 0.82)	1.64 (1.49 - 1.79)	1.46 (1.31 - 1.62)	1.80 (1.60 - 1.99)	2.51 (2.25 - 2.78)	3.22 (2.85 - 3.60)
Black	0.72 (0.58 - 0.87)	2.25 (1.99 - 2.51)	1.93 (1.65 - 2.20)	1.59 (1.31 - 1.88)	2.75 (2.31 - 3.18)	4.04 (3.38 - 4.70)
Any other Asian	0.67 (0.43 - 0.91)	1.72 (1.33 - 2.11)	1.43 (1.03 - 1.83)	1.52 (1.06 - 1.98)	2.79 (2.06 - 3.51)	3.15 (2.18 - 4.11)
Other	0.92 (0.54 - 1.29)	1.59 (1.08 - 2.09)	2.54 (1.81 - 3.26)	1.68 (0.98 - 2.38)	2.42 (1.43 - 3.42)	3.19 (1.78 - 4.59)
Mixed	0.73 (0.28 - 1.17)	0.88 (0.37 - 1.39)	2.14 (1.25 - 3.03)	2.43 (1.29 - 3.57)	3.23 (1.65 - 4.81)	2.56 (0.81 - 4.30)
Chinese	0.66 (0.04 - 1.27)	1.32 (0.42 - 2.23)	1.68 (0.53 - 2.83)	0.46 (-0.25 - 1.17)	2.68 (0.73 - 4.63)	0.00 (0.00 - 0.00)
Unknown	0.87 (0.20 - 1.54)	3.01 (1.56 - 4.45)	2.05 (0.42 - 3.69)	4.15 (0.88 - 7.41)	2.56 (-0.97 - 6.10)	3.33 (-2.06 - 8.73)
**Baseline DR grade**						
No retinopathy	0.18 (0.15 - 0.21)	0.60 (0.54 - 0.66)	0.68 (0.61 - 0.75)	0.91 (0.81 - 1.00)	1.48 (1.34 - 1.62)	2.13 (1.92 - 2.35)
Retinopathy in one eye	1.20 (0.97 - 1.44)	3.41 (3.01 - 3.81)	3.65 (3.19 - 4.11)	3.94 (3.39 - 4.50)	5.32 (4.57 - 6.07)	6.76 (5.68 - 7.83)
Retinopathy in both eyes	6.87 (6.13 - 7.61)	16.72 (15.56 - 17.88)	13.99 (12.70 - 15.28)	12.56 (11.07 - 14.04)	14.96 (13.03 - 16.90)	13.61 (11.20 - 16.03)

Supplementary table 4. Incidence rates of any diabetic retinopathy (people with R0M0 at first screen, n = 107,701) and sight-threatening diabetic retinopathy (people with R0M0 and R1M0 at first screen, n = 137,591) per 100 person-years by ethnic group.

Ethnicity	Any DR IR (95% CI)	STDR IR (95% CI)
White	7.78 (7.78 - 7.78)	1.77 (1.77 - 1.77)
South Asian	8.12 (8.12 - 8.12)	2.44 (2.44 - 2.44)
Black	8.04 (8.03 - 8.04)	2.68 (2.68 - 2.68)
Any other Asian	7.48 (7.47 - 7.48)	2.24 (2.24 - 2.25)
Other	8.38 (8.36 - 8.39)	2.33 (2.32 - 2.33)
Mixed	7.66 (7.63 - 7.70)	2.41 (2.40 - 2.43)
Chinese	8.06 (8.00 - 8.12)	1.65 (1.63 - 1.67)
Unknown	9.33 (9.29 - 9.36)	2.61 (2.59 - 2.62)

BMJ Publishing Group Limited (BMJ) disclaims all liability and responsibility arising from any reliance placed on this supplemental material which has been supplied by the author(s)

Supplementary table 5. Cumulative incidence rate of any diabetic retinopathy per 100 person-years by follow-up (95% confidence intervals).

Characteristic	Year 1	Year 2	Year 3	Year 4	Year 5	Year 10
**Overall**	3.64 (3.54 - 3.73)	6.37 (6.24 - 6.49)	6.97 (6.84 - 7.09)	7.22 (7.09 - 7.35)	7.47 (7.34 - 7.60)	7.95 (7.82 - 8.09)
**Age groups**						
Less than 45 years	3.31 (3.11 - 3.50)	6.00 (5.74 - 6.26)	6.70 (6.43 - 6.98)	7.16 (6.88 - 7.44)	7.60 (7.31 - 7.89)	8.51 (8.20 - 8.81)
45 to <55 years	3.42 (3.23 - 3.60)	6.10 (5.86 - 6.35)	6.69 (6.44 - 6.95)	6.94 (6.68 - 7.20)	7.19 (6.93 - 7.45)	7.80 (7.53 - 8.08)
55 to <65 years	3.63 (3.44 - 3.82)	6.10 (5.86 - 6.35)	6.61 (6.36 - 6.86)	6.77 (6.52 - 7.03)	6.97 (6.72 - 7.23)	7.32 (7.06 - 7.59)
65 years and over	4.05 (3.87 - 4.23)	7.05 (6.82 - 7.29)	7.67 (7.43 - 7.91)	7.87 (7.62 - 8.11)	8.04 (7.79 - 8.29)	8.27 (8.02 - 8.52)
**Sex**						
Female	3.50 (3.37 - 3.63)	6.16 (5.98 - 6.33)	6.80 (6.62 - 6.99)	7.03 (6.85 - 7.22)	7.30 (7.11 - 7.48)	7.82 (7.62 - 8.01)
Male	3.76 (3.63 - 3.89)	6.56 (6.39 - 6.73)	7.12 (6.94 - 7.29)	7.39 (7.20 - 7.57)	7.63 (7.45 - 7.81)	8.08 (7.89 - 8.27)
**Ethnicity**						
White	3.87 (3.71 - 4.03)	6.46 (6.26 - 6.67)	7.06 (6.85 - 7.27)	7.24 (7.02 - 7.45)	7.43 (7.21 - 7.65)	7.78 (7.56 - 8.00)
South Asian	3.25 (3.10 - 3.40)	6.21 (6.01 - 6.42)	6.91 (6.70 - 7.13)	7.19 (6.97 - 7.41)	7.51 (7.29 - 7.74)	8.12 (7.89 - 8.35)
Black	3.92 (3.68 - 4.16)	6.49 (6.19 - 6.80)	6.97 (6.65 - 7.29)	7.27 (6.95 - 7.60)	7.49 (7.16 - 7.82)	8.04 (7.70 - 8.38)
Any other Asian	3.48 (3.09 - 3.87)	6.04 (5.54 - 6.55)	6.49 (5.97 - 7.01)	6.85 (6.31 - 7.39)	7.03 (6.49 - 7.57)	7.48 (6.92 - 8.03)
Other	4.07 (3.49 - 4.65)	6.45 (5.72 - 7.17)	6.92 (6.17 - 7.67)	7.43 (6.65 - 8.20)	7.83 (7.03 - 8.62)	8.38 (7.56 - 9.20)
Mixed	3.01 (2.26 - 3.77)	5.85 (4.81 - 6.89)	6.17 (5.10 - 7.24)	6.45 (5.36 - 7.55)	6.79 (5.68 - 7.91)	7.66 (6.48 - 8.85)
Chinese	3.79 (2.64 - 4.94)	7.09 (5.54 - 8.64)	7.54 (5.95 - 9.13)	7.81 (6.19 - 9.43)	8.20 (6.54 - 9.85)	8.06 (6.42 - 9.70)
Unknown	4.12 (3.29 - 4.95)	7.82 (6.70 - 8.94)	9.05 (7.85 - 10.24)	9.08 (7.88 - 10.28)	9.19 (7.99 - 10.40)	9.33 (8.11 - 10.54)

Supplementary table 6. Semi-parametric multivariable Cox proportional hazards model for interval censored data. Hazard ratios (HR) are mutually adjusted for all factors shown in the table. HR greater than 1 imply greater hazards for vision-threatening diabetic retinopathy (DR). Bold p-values show statistically significant results.

Characteristic	HR	95% CI	p-value
**Age (per 5-year increase)**	0.92	(0.91 - 0.93)	**$7.5x10^{-129}$**
**Age groups**			
<45 years	1.00		
45 to <55 years	0.80	(0.75 - 0.84)	**$5.1x10^{-23}$**
55 to <65 years	0.61	(0.56 - 0.66)	**$9.7x10^{-82}$**
65 years and over	0.56	(0.50 - 0.61)	**$4.2x10^{-112}$**
**Sex**			
Female	1.00		
Male	1.04	(1.00 - 1.07)	**0.028**
**Baseline DR grade**			
No retinopathy	1.00		
Retinopathy in both eyes	8.00	(7.96 - 8.05)	**$3.5x10^{-2209}$**
Retinopathy in one eye	3.07	(3.03 - 3.11)	**$1.0x10^{-632}$**
**Ethnicity**			
White	1.00		
South Asian	1.37	(1.33 - 1.41)	**$1.9x10^{-51}$**
Black	1.58	(1.53 - 1.63)	**$9.1x10^{-76}$**
Any other Asian	1.26	(1.18 - 1.34)	**$1.4x10^{-09}$**
Other	1.30	(1.2 - 1.39)	**$1.2x10^{-07}$**
Mixed	1.38	(1.23 - 1.53)	**$1.9x10^{-05}$**
Chinese	0.98	(0.75 - 1.22)	0.888
Unknown	1.38	(1.16 - 1.59)	**0.003**
**Duration of diabetes (per 5 years)**	1.15	(1.14 - 1.16)	**$3.3x10^{-136}$**
**Type of diabetes**			
Type 2	1.00		
Type 1	1.02	(0.94 - 1.11)	0.594
Other	0.71	(0.19 - 1.24)	0.198
Missing	1.25	(1.12 - 1.37)	**$5.5x10^{-04}$**
**IMD**			
1 (Most deprived)	1.00		
2	1.04	(0.98 - 1.10)	0.239
3	1.02	(0.96 - 1.08)	0.444
4	0.98	(0.92 - 1.05)	0.586
5 (least deprived)	0.93	(0.85 - 1.01)	0.064

[1]HR = Hazard Ratio, CI = Confidence Interval

Supplementary table 7. Multivariable Cox proportional hazards model for right censored data with development of any diabetic retinopathy (DR) as outcome of interest. Hazard ratios (HR) are mutually adjusted for all factors shown in the table. HR greater than 1 imply greater hazards for DR. Bold p-values show statistically significant results.

Characteristic	HR[1]	95% CI[1]	p-value
**Age (per 5-year increase)**	0.99	0.98, 0.99	**$2.4x10^{-12}$**
**Age categories**			
<45yr	1.00		
45 to <55yr	0.90	0.87, 0.93	**$3.9x10^{-11}$**
55 to <65yr	0.81	0.78, 0.84	**$4.8x10^{-39}$**
65yr and over	0.87	0.84, 0.90	**$6.8x10^{-18}$**
**Sex**			
Female	1.00		
Male	1.06	1.03, 1.08	**$1.5x10^{-07}$**
**Ethnicity**			
White	1.00		
South Asian	1.03	1.00, 1.06	**0.021**
Black	1.03	1.00, 1.06	0.070
Any other Asian	0.96	0.92, 1.01	0.096
Other	1.09	1.02, 1.16	**0.006**
Mixed	1.03	0.94, 1.13	0.550
Chinese	1.06	0.94, 1.20	0.318
Unknown	1.36	1.21, 1.54	**$5.7x10^{-07}$**
**Duration of diabetes (per 5-year increase)**	1.18	1.17, 1.19	**$1.1x10^{-377}$**
**Type of diabetes**			
Type 2	1.00		
Type 1	1.36	1.28, 1.44	**$2.5x10^{-26}$**
Other	1.01	0.77, 1.32	0.951
Missing	1.25	1.16, 1.35	**$7.6x10^{-09}$**
**Deprivation (IMD quintiles)**			
1	1.00		
2	0.99	0.96, 1.03	0.692
3	0.98	0.95, 1.02	0.338
4	0.98	0.94, 1.02	0.318
5	0.97	0.93, 1.01	0.152
Missing	1.78	1.05, 3.02	**0.032**

[1]HR = Hazard Ratio, CI = Confidence Interval
